# Effect of Poly (Caprolactone) Introduction Site on the Network Structure and Properties of Glycidyl Azide Polymer Adhesive

**DOI:** 10.3390/polym17050661

**Published:** 2025-02-28

**Authors:** Chengzhao Tu, Zhengyuan Wang, Fengdan Zhu, Dengsheng Yang, Chang Liu, Chaofei Bai, Guoping Li, Yunjun Luo

**Affiliations:** School of Materials Science and Engineering, Beijing Institute of Technology, Beijing 100081, China

**Keywords:** GAP, blending, crystallize, mechanical property, adhesive

## Abstract

Copolymers of glycidyl azide polymer (GAP) and poly (caprolactone) (PCL) were obtained by introducing PCL molecular chains at both ends or side groups of GAP molecular chains, respectively. GAP/PCL elastomers were prepared via polyurethane curing reaction and compared with GAP/PCL elastomers prepared by physical blending, in order to clarify the relationship between microstructure and macroscopic properties. The results showed that no GAP and PCL phase separation was observed in the chemically bonded GAP/PCL elastomers. The elongation at break of the thermosetting GAP/PCL block copolymer elastomer increased significantly from 268% to 300% due to the increase in molecular weight between crosslinking points. The GAP/PCL graft copolymer, with its longer PCL segment length and higher segment mobility, formed microcrystalline domains within the elastomer, resulting in a significant improvement in tensile strength from 0.32 MPa to 1.07 MPa. In addition, differential scanning calorimetry (DSC) and thermogravimetric analysis (TGA) revealed that the glass transition temperature of the GAP/PCL elastomer was 2.6 °C lower than that of the pure GAP elastomer, and the thermal stability was also enhanced.

## 1. Introduction

Glycidyl azide polymer (GAP) exhibits several advantageous properties, including a positive heat of formation [1,2,3], high density [4], low mechanical sensitivity [5,6,7], and excellent thermal stability [8,9,10,11]. Its gas cleanliness and low characteristic signature further contribute to its suitability as a binder for the preparation of high-energy, insensitive, and low-signature propellants [12,13]. However, the rigidity of the azidomethyl side group restricts the flexibility of the GAP molecular chain, leading to an elevated glass transition temperature [14,15]. Moreover, the bulky azidomethyl side group reduces the number of carbon atoms in the GAP main chain, resulting in weaker intermolecular forces compared to other polyethers of similar molecular weight. This, in turn, contributes to the compromised mechanical properties observed in GAP-based binder systems [16,17].

Blending with flexible-chain polymers is a common strategy to enhance the mechanical properties of GAP-based binder systems [18,19,20,21]. Byoung et al. [22] incorporated a blend of flexible poly(ethylene glycol) (PEG) and PCL into the binder formulation. Their results indicated that PCL exhibited a greater enhancement in the mechanical and thermal properties of GAP compared to PEG, and the GAP/PCL system could accommodate a higher plasticizer content than the GAP/PEG system. At lower plasticizer ratios, a decrease in the glass transition temperature (Tg) was observed with increasing PCL content. Min et al. [23] found that the introduction of PCL into the GAP matrix could enhance intermolecular interactions within the network, thereby increasing stress. Furthermore, it increased the effective length between molecular chains, improving the elongation at break of the propellant. However, excessive PCL content (>30%) could lead to crystallization, resulting in a sudden increase in stress and an excessively high initial modulus.

However, the disparate polarities between GAP and other macromolecules often lead to compatibility issues and susceptibility to phase separation, significantly limiting their practical applications [24]. To address this challenge, many researchers have initially combined GAP with flexible-chain polymers via diisocyanates to synthesize block copolymers, which are subsequently subjected to curing and crosslinking processes [11,19,22,23,25]. Nevertheless, this approach also presents several challenges. First, the synthesis of block copolymers typically requires the use of excess isocyanates, potentially leading to undesirable side reactions [26,27,28]. Second, the fixed molecular weights of the raw materials make it difficult to precisely control the GAP to flexible-chain polymer ratio in the resulting block copolymers. Third, block copolymers can be viewed as GAP chains extended at their termini, potentially increasing binder viscosity and significantly reducing the processing performance of propellant slurries [26,27,28].

Currently, research on GAP primarily focuses on the selection of mixture components, with relatively limited exploration of mixing methods. In this study, we synthesized and characterized GAP/PCL block copolymers and GAP/PCL graft copolymers, examining the structures and properties of these two prepolymers, GAP/PCL blends, and the elastomers obtained by curing them with N-100. The objective was to determine the effects of different PCL blending methods on the structure and properties of GAP-based binder systems.

## 2. Experimental

### 2.1. Materials

GAP (Mn = 3704, OH index: 0.54 mmol/g) and Desmodur (N-100, NCO index: 5.38 mmol/g) were supplied by Liming Research Institute of Chemical Industry, Henan, China; PCL (Mn = 2000, hydroxyl value: 56.11 mg KOH/g) was purchased from Juren Chemical Hitechnology Co., Ltd., Yueyang, China. 3-butyn-1-ol (3-Butyn-1-ol, >99%), ε-caprolactone (ε-CL, >99%), Stannous octanoate (Sn(Oct)_2_, >99%) and dioctyl sebacate (DOS, >99%), were purchased from Meryer Biochemical Technology Co., Ltd., Shanghai, China. Dibutyltin dilaurate (T12, >99%) and triphenylbismuth (TPB, >99%) were purchased from Beijing Mreda Technology Co., Ltd. and configured as a 5% DOS solution before use.

### 2.2. Synthesis of GAP/PCL Block Copolymer (PCL-b-GAP-b-PCL)

A catalytic amount of Sn(Oct)_2_ and 40 g GAP were added to the flask. Thereafter, 10 g ε-CL was introduced, and the reaction was conducted under nitrogen protection at 120 °C for 6 h. The product was dissolved in trichloromethane, precipitated with ice ethanol, and dried in an oven at 60 °C for 12 h to obtain the PCL-b-GAP-b-PCL.

### 2.3. Synthesis of GAP/PCL Graft Copolymer (GAP-g-PCL)

An amount of 0.72 g of 3-butyne-1-ol, 20 g of ε-CL and a catalytic amount of Sn(OCT)_2_ were added into the flask and reacted for 6 h at 120 °C under the protection of nitrogen. The product was dissolved in chloroform, precipitated with cold ethanol, and oven at 60 °C overnight to obtain alkynyl polycaprolactone (Alk-PCL). Amounts of 5 g Alk-PCL and 20 g GAP were added into the flask and reacted at 100 °C for 6 h to obtain the GAP-g-PCL.

### 2.4. Preparation of GAP and GAP/PCL Elastomer

**Figure 1:**
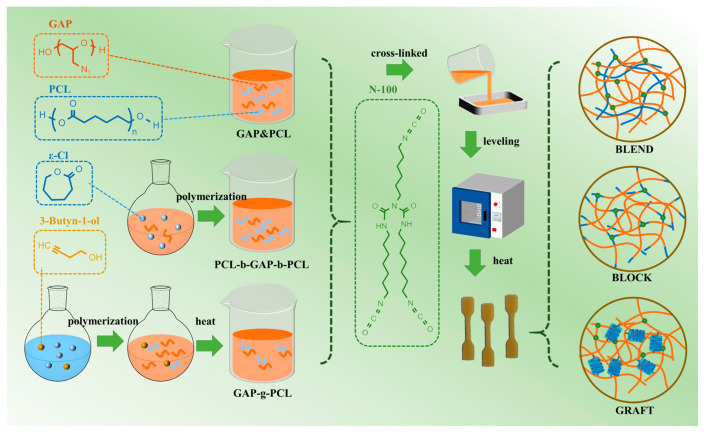
Schematic diagram for preparing prepolymers and elastomers.

The preparation method refers to our previous work [20]. The calculated amount of gap or gap/pcl prepolymer, N-100 and catalyst (the mass ratio of TPB to T12 is 3:1) were added into the beaker and stirred at 60 °C until the components were completely mixed. After removing the bubbles under vacuum, this was slowly poured into the PTFE mold. When the mixing flow in the mold was flat, it was transferred to a constant temperature oven at 60 °C and cured for 7 days. The network structure of the prepared elastomer is shown in Figure 1.

### 2.5. Characterizations

The molecular weights were obtained using gel permeation chromatography (GPC). The sample concentration was 10 mg/mL~15 mg/mL, and the testing temperature was 40 °C. Chromatographic pure tetrahydrofuran was used as the mobile phase, with a flow rate of 1.0 mL/min. A fitting curve was established using monodisperse polystyrene standard.

The viscosity of the prepolymer was measured using an R/S-SST Plus rheometer (Brookfield, USA) with a propeller rotor of 20–10 mm. The viscosity test used controlled shear rate mode (CSR), with a test temperature of 60 °C and a shear rate of 1 s^−1^. A data point was collected every 0.5 s for 60 s, and each sample was independently prepared and tested 5 times to eliminate operational errors and batch differences.

The ^1^H nuclear magnetic resonance (^1^H NMR) spectra of the samples were measured by a Bruker 400 MHz NMR spectrometer in chloroform-d (CDCl_3_). Tetramethylsilane (TMS) was used as a reference.

Fourier transform infrared spectroscopy (FT-IR) was measured by Nicolet iS5 (Thermo Fisher, USA). ATR total reflection mode was selected with 48 scanning times and a resolution of 4 cm^−1^.

The mechanical properties were determined by the electronic universal testing machine (Instron 5966, USA). The test method refers to GB/T 528-1998. The spline was cut into dumbbell shape, the test temperature was 25 °C, and the tensile rate was 100 mm/min. Each sample was tested 5 times and the average value was taken.

X-ray diffraction (XRD) analysis and Small-Angle X-ray Scattering (SAXS) were carried out using an X-ray diffractometer (Bruker D8, Advance, Germany), which was equipped with a Cu Kα source and worked in the 2θ range of 5–50 at 40 kV and 30 mA.

DSC (differential scanning calorimetry) was measured by DSC/500 (Mettler Toledo, Switzerland). The mass of the sample was 5~10 mg, which increased from room temperature to 100 °C, then decreased to −80 °C after holding for 3 min, and then increased to 20 °C. The heating rate was 10 °C/min, the atmosphere was nitrogen, and the flow rate was 40 mL/min.

Low-field nuclear magnetic resonance (LF-NMR) was measured using VTMR20-010 V-T (Niumai Corporation, China). The elastomer was chopped and placed in a 1 mL NMR bottle for testing at a temperature of 30 °C. Each sample was tested 5 times, and the average value was taken.

Dynamic thermal mechanical properties (DMA) were assessed on a DMA/SDTA861e (Mettler Toledo, Switzerland). The temperature range was −60 to 90 °C, the heating rate was 5 °C/min, and the frequency was 10 Hz.

The fractured morphologies were characterized by a JSM-5800 scanning electron microscope (SEM, Hitachi, Japan). The sample was plated with platinum at 10 mA for 40 s, and then tested under vacuum.

The phase structure was measured by atomic force microscopy (AFM) (Bruker Dimension ICON, Germany). The surface of the sample was smooth, with dimensions of 30 mm × 5 mm, and the patterns were morphology and phase diagram.

Thermogravimetric analysis—Fourier transform infrared spectroscopy (TGA—FTIR) was measured using a Nicolet iS50 (ThermoFisher, Waltham, MA, USA). The sample weight was approximately 1.5 mg. The atmosphere was nitrogen with a flow rate of 40 mL/min. The temperature range was from 50 °C to 600 °C, and the heating rate was 10 °C/min. The number of infrared scans was 4, and the resolution was 2 cm^−1^.

## 3. Results and Discussion

### 3.1. Synthesis and Characterization of GAP/PCL Prepolymers

#### 3.1.1. Synthesis and Characterization of PCL-b-GAP-b-PCL

**Figure 2:**
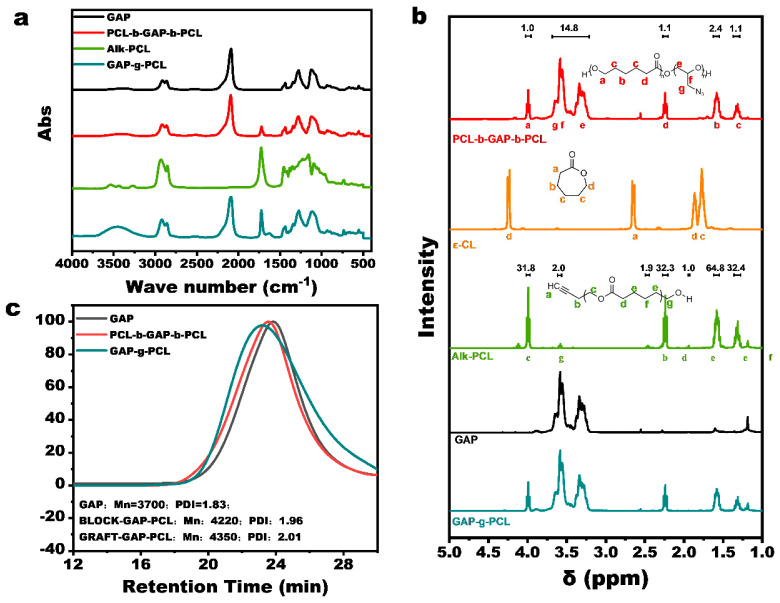
(**a**) Infrared spectra of PCL-b-GAP-b-PCL, GAP-g-PCL, and their reaction materials; (**b**) H-NMR spectra of PCL-b-GAP-b-PCL and reaction materials; (**c**) GPC results of PCL-b-GAP-b-PCL, GAP-g-PCL, and their reaction materials.

The synthetic route for PCL-b-GAP-b-PCL is illustrated in Figure S1a. Ring-opening polymerization of ε-CL, catalyzed by stannous octanoate and using GAP as the macromolecular initiator, resulted in a reaction yield of approximately 91%. Figure 2a shows the infrared spectrum of PCL-b-GAP-b-PCL. Compared to GAP, the infrared spectrum of PCL-b-GAP-b-PCL exhibits a characteristic stretching vibration peak at approximately 1720 cm^−1^, corresponding to the carbon–oxygen double bond of the ester group in the PCL segment. This observation confirms the successful completion of the polymerization process. Furthermore, GPC analysis revealed a single peak, rather than multiple peaks, indicating that the generated PCL polymerized uniformly from the termini of GAP, providing additional evidence for the successful synthesis of the target block copolymer. GPC analysis indicated that the PCL segment constituted approximately 20% of the mass of PCL-b-GAP-b-PCL, consistent with the expected ratio based on the initial feed. Figure 2b depicts the ^1^H NMR spectra of PCL-b-GAP-b-PCL, hydroxyl PCL, and GAP, with peak assignments for PCL-b-GAP-b-PCL provided in the accompanying illustration. Comparison of the three spectra reveals that the ^1^H NMR spectrum of PCL-b-GAP-b-PCL is essentially a superposition of the ^1^H NMR spectra of PCL and GAP. Ultimately, calculation of the peak areas determined the actual mass ratio of PCL in the system to be 18.1%.

#### 3.1.2. Synthesis and Characterization of GAP-g-PCL

The synthetic route for GAP-g-PCL is depicted in Figure S1b. Initially, alkynyl-terminated PCL (Alk-PCL) was synthesized via polymerization of ε-CL with butynol as the initiator. Subsequently, Alk-PCL was grafted onto the GAP chain through a reaction between the alkynyl and azide groups. Figure S2 shows that the infrared spectrum of Alk-PCL is similar to that of hydroxy-PCL, but exhibits a characteristic alkynyl peak at 3245 cm^−1^. Comparison of the ^1^H NMR spectra of ε-caprolactone and Alk-PCL in Figure 2c reveals that the ring-opening polymerization of ε-caprolactone reduces the shielding effect, causing the proton peaks to shift upfield due to the loss of magnetic anisotropy of the π bond. In the ^1^H NMR spectrum of Alk-PCL, the ε-caprolactone peak was essentially undetectable, while a characteristic alkynyl proton peak was observed at δ = 2.46. These IR and ^1^H NMR results confirm the successful synthesis of Alk-PCL. Based on ^1^H NMR peak area calculations, the molecular weight of the product was determined to be approximately 1910.

The synthesized Alk-PCL and GAP were heated at 75 °C for 24 h, and samples were collected at 0 h, 12 h, and 24 h for ^1^H NMR analysis. The results are presented in Figure S3. As the reaction progressed, the signal at δ = 1.94, corresponding to the α-proton of the alkynyl group, gradually diminished, while a new signal appeared at δ = 1.78. This is attributed to the formation of a triazole structure adjacent to the proton, which enhances conjugation and increases shielding compared to the original alkynyl group. Furthermore, the characteristic alkynyl hydrogen peak disappeared in the infrared spectrum of the product. These results strongly support the successful preparation of GAP with a PCL graft-modified structure.

### 3.2. Characterization of GAP/PCL Prepolymer Properties

During the preparation of solid propellants, slurry viscosity serves as a crucial indicator of product quality. Excessively high viscosity hinders the casting process, while excessively low viscosity leads to settling of the solid filler and subsequent delamination. GAP-based propellants, in particular, are characterized by rigid side groups along the main chain, which limits molecular chain flexibility. Consequently, slurry viscosity significantly influences their practical application. Therefore, determining the viscosity of the prepolymer is essential before investigating the characteristics of the thermoset elastomers.

**Figure 3:**
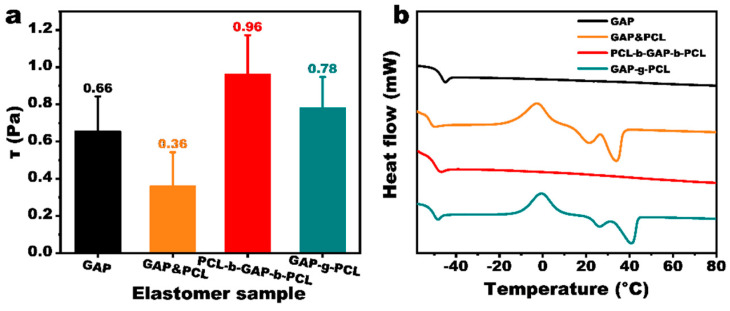
(**a**) Viscosity of GAP and GAP/PCL prepolymers; (**b**) DSC spectra of GAP/PCL prepoly-mer.

Considering that the curing temperature for propellants is typically 60 °C, viscosity tests were performed on PCL-b-GAP-b-PCL and GAP-g-PCL at this temperature. Simultaneously, a control sample (GAP&PCL) was prepared by blending GAP with PCL (number average molecular weight of 2000 and terminal hydroxyl groups) in an 8:2 mass ratio, and the results are shown in Figure 3a. At 60 °C, the viscosity of pure GAP was 0.66 Pa·s. The viscosity of GAP&PCL decreased to 0.36 Pa·s due to the flexible PCL chains entering the intermolecular space of GAP, which reduced the overall system viscosity. However, the viscosity of PCL-b-GAP-b-PCL increased to 0.96 Pa·s. This is because the PCL chain segments at both ends effectively extended the GAP main chain, resulting in a higher molecular weight. Furthermore, because PCL is evenly distributed at both ends of the GAP macromolecules, each PCL chain segment contains fewer chain links, limiting its mobility and reducing its lubricating effect compared to GAP&PCL. The viscosity of the GAP-g-PCL prepolymer also increased, but to a lesser extent than that of the PCL-b-GAP-b-PCL prepolymer. This is attributed to the PCL in the GAP-g-PCL prepolymer existing as side chains, which increases the spacing between the rigid GAP chain segments. Therefore, GAP-g-PCL has better process performance than PCL-b-GAP-b-PCL.

The thermal properties of the prepolymers were characterized using DSC, and the results are shown in Figure 3b. Crystallization and melting peaks, characteristic of the crystalline polymer PCL, were observed in GAP&PCL and GAP-g-PCL, but not in PCL-b-GAP-b-PCL. This suggests that the PCL chain segments at the termini of GAP are too short to exhibit crystalline behavior. The crystallization temperatures of GAP&PCL and GAP-g-PCL were similar; however, the crystal melting temperature of GAP-g-PCL was 5 °C higher than that of GAP&PCL. This indicates that the PCL crystalline regions in GAP-g-PCL are more ordered. Although the crystallization temperatures of GAP&PCL and GAP-g-PCL showed minimal difference, the melting temperature of the crystals in GAP-g-PCL was 5 °C higher than that of GAP&PCL, implying that the PCL crystalline region within GAP-g-PCL may exhibit greater regularity, potentially due to stronger hydrogen bonding.

### 3.3. Network Structure of GAP/PCL Elastomers

**Figure 4:**
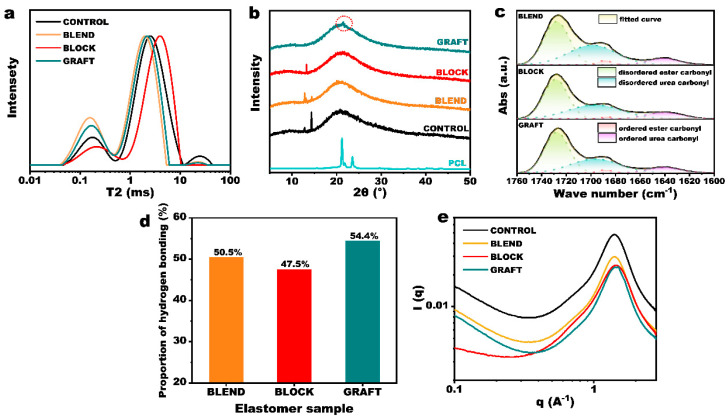
Characterization of GAP/PCL elastomers. (**a**) LF-NMR curves, (**b**) X-ray diffraction curves, (**c**) FT-IR curves and (**d**) the corresponding hydrogen bond statistics, (**e**) SAXS images.

The above prepolymers were cured with N-100 to obtain thermosetting elastomers (elastomer information is provided in Table S1). The transverse spin–spin relaxation time (T2) of hydrogen atoms in the three elastomers was determined using LF-NMR, and the inversion image is presented in Figure 4a. A smaller T2 value indicates a more restricted motion of hydrogen atoms. The LF-NMR spectra of the elastomers generally show three peaks, corresponding to hydrogen protons in (1) hard domains forming hydrogen bonds or crystals, (2) soft domains, and (3) small molecules, primarily unreacted curing agents. Notably, BLOCK elastomers exhibited negligible small molecule peaks, and the small molecule peaks of BLEND and GRAFT elastomers were also significantly lower than those of the CONTROL elastomer, indicating that the incorporation of PCL enhances the curing reaction efficiency. This phenomenon can be attributed to the higher reactivity of the terminal hydroxyl group of PCL compared to that of GAP. The terminal hydroxyl group of PCL is a primary hydroxyl group, whereas the two terminal hydroxyl groups of GAP include a secondary hydroxyl group. Although the other hydroxyl group of GAP is a primary hydroxyl group, it is connected to an azido methylene group at the β position, while the terminal hydroxyl group of PCL is connected to five methylene groups. This endows PCL with greater reactivity, lower steric hindrance, and a higher propensity to participate in the curing reaction.

Comparing the ratios of the three peak areas reveals that the hard domain content in the elastomers, in descending order, is BLEND > GRAFT > BLOCK. This trend correlates with the hydroxyl values of the three prepolymers, which also decrease in the same order. Hydrogen bonding in elastomers is formed between the active hydrogen in the urethane group and the oxygen in the carbonyl group. Urethane linkages are formed from the reaction of hydroxyl and isocyanate groups. Therefore, at a constant R value (ratio of isocyanate to hydroxyl groups), a higher hydroxyl functionality leads to a greater number of urethane groups in the cured elastomer. This, in turn, results in increased hydrogen bonding and a corresponding expansion of the hard domain area.

Carbonyl groups serve as the primary acceptors of amino protons. Upon hydrogen bond formation, the carbon–oxygen double bond length increases, and the bond energy decreases, resulting in a shift of the FT-IR absorption peak to lower wavenumbers. Figure S4 shows the FT-IR spectra of the three GAP/PCL elastomers. Because the types and proportions of functional groups are nearly identical in the three systems, their infrared spectra are very similar. The carbonyl peak region spans 1660–1760 cm^−1^, encompassing contributions from unassociated ester carbonyl (1728 cm^−1^), associated ester carbonyl (1688 cm^−1^), unassociated urea carbonyl (1696 cm^−1^), and associated urea carbonyl (1640 cm^−1^). Peak fitting results for this region are presented in Figure 4c. Figure 4d shows the percentage of carbonyl groups involved in hydrogen bonding in each elastomer, as determined from peak areas. This reflects the relative ease of hydrogen bond formation within the three molecular chain structures, which is related to chain segment mobility. While the proportion of hydrogen bonds varies slightly among the three elastomers, they all constitute approximately 50% of the total carbonyl groups. Therefore, the hard domain volume formed by hydrogen bonding is primarily determined by the prepolymer functionality.

Because PCL is a crystalline polymer, its molecular chains are relatively ordered, allowing for crystallization and the formation of hard domains. To further investigate the origin of hard segment composition in each elastomer, XRD was performed. Figure 4c shows that, among the four elastomers, only the GRAFT elastomer exhibits crystallinity. Interestingly, although the GAP&PCL blend exhibits melting peaks in Figure 2c, it does not show any diffraction peaks in the XRD spectra after curing. This is likely because both ends of the PCL chains are fixed in the blend elastomer, hindering the regular arrangement of chain segments. To promote crystallization in this system, the molecular weight and mass fraction of PCL would need to be increased to enhance the length and proportion of mobile chain segments between crosslinks. As shown in Figure S5, an elastomer with 30% PCL (molecular weight = 3000) exhibits a crystallization peak. In the GRAFT elastomer, the wider molecular chain spacing enhances chain segment mobility compared to the blend, facilitating crystal formation through ordered arrangement.

Analysis of the normalized soft segment region characteristic peaks (Figure 4a), in conjunction with crosslinking density measurements (Figure S6), reveals a shift towards shorter relaxation times in the BLEND and GRAFT elastomers compared to the GAP elastomer. This indicates that the introduction of hard segments promotes the formation of physical crosslinking points, leading to an increased crosslinking density and a concomitant reduction in the overall mobility of the soft segment regions. In contrast, the BLOCK elastomer exhibits significantly longer soft segment relaxation times compared to the other three elastomers. This observation can be attributed not only to a lower density of physical crosslinking points, but also to a reduced chemical crosslinking density resulting from the lower functionality of the prepolymer. Furthermore, the chemical crosslinking points in the BLOCK elastomer are predominantly surrounded by flexible PCL chains, which likely contributes to the enhanced overall chain mobility observed in the soft segment regions.

The SAXS results (Figure 4e) reveal a scattering peak at 1.4 Å^−1^ in both the GAP and GAP&PCL elastomers, indicating the presence of a microphase-separated structure. This observation aligns with the low-field NMR analysis, which also suggests the presence of two distinct phases: a hard segment and a soft segment. Notably, the GRAFT elastomer exhibited an additional, albeit small, scattering peak at 1.5 Å^−1^, corresponding to the (110) crystallographic plane of the PCL crystalline region (d-spacing ≈ 4.2 nm).

**Figure 5:**
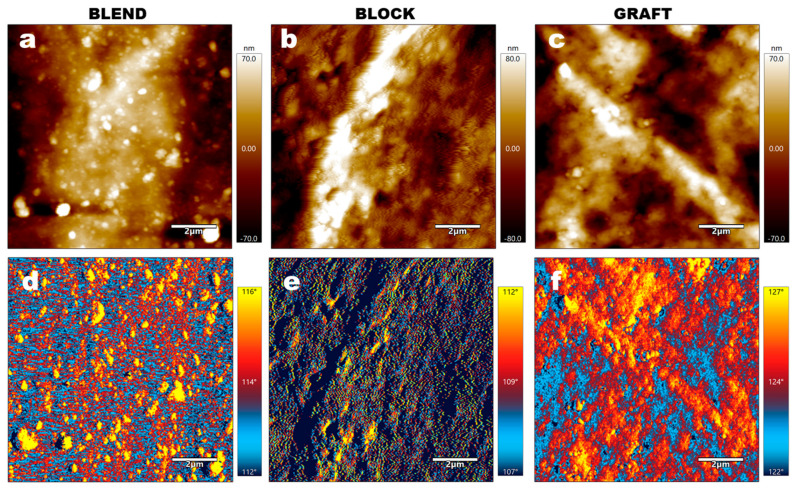
AFM images of elastomer samples: (**a**–**c**) Topography images and (**d**–**f**) phase images. (**a**,**d**) BLEND elastomer; (**b**,**e**) BLOCK elastomer; (**c**,**f**) GRAFT elastomer.

To further characterize the microphase separation behavior, AFM was performed on the three elastomers, with the results shown in Figure 5. In the BLEND elastomer, PCL domains are observed as islands dispersed within the GAP matrix. This heterogeneous structure is likely to influence the mechanical properties of the material. In contrast, the BLOCK elastomer exhibits a more homogeneous morphology, with the PCL phase no longer appearing as discrete islands within the GAP. This homogeneity is attributed to the covalent bonding of PCL chain segments to both ends of the GAP block in the PCL-b-GAP-b-PCL precursor. Furthermore, the relatively short length of the PCL chains hinders their aggregation via intermolecular forces, preventing the formation of large PCL-rich phases. Although PCL is also covalently bonded to GAP in the GAP-g-PCL precursor, the AFM images reveal the presence of bright, PCL-enriched regions within the GRAFT elastomer. This observation is attributed to the longer PCL chain segments in GRAFT compared to BLOCK, coupled with the enhanced mobility of the PCL chains facilitated by the comb-like architecture. These factors promote aggregation and potentially even crystallization. However, similarly to the PCL-b-GAP-b-PCL system, the homogeneous bonding of PCL chains to the GAP matrix in GRAFT results in a significant difference in the morphology of the PCL-rich regions compared to the BLEND elastomer. Instead of existing as isolated, island-like structures, as observed in BLEND, the PCL-rich domains in GRAFT form a continuous, interconnected phase.

### 3.4. Static Mechanical Properties of GAP/PCL Elastomers

**Figure 6:**
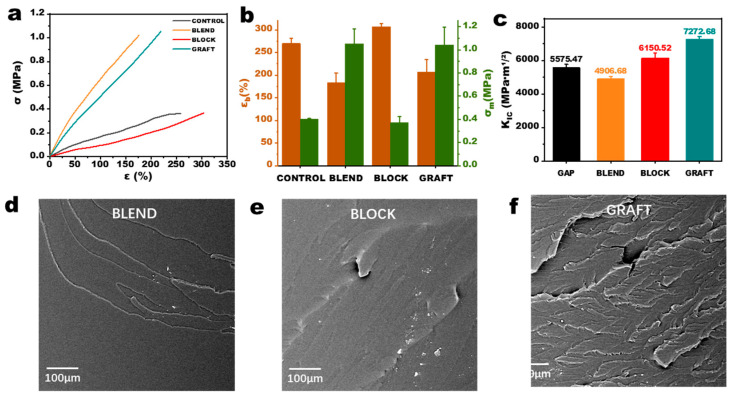
Characterization of GAP/PCL elastomers. (**a**) Tensile curves, (**b**) parameter statistics, (**c**) critical stress intensity factor and (**d**–**f**) fractography analysis.

The tensile strength of the binder directly influences the propellant grain’s ability to withstand various loads. Propellants with higher tensile strength exhibit improved resistance to complex loading conditions encountered during manufacturing, storage, transportation, and flight, such as impact, vibration, acceleration, pressure, and thermal stresses [29]. The static tensile test results for the elastomers are shown in Figure 6a,b. Compared to GAP elastomers, the elongation at break of BLOCK elastomers increased from 250% to 300% due to the incorporation of more flexible PCL chain segments and the extension of the prepolymer main chain. However, the maximum tensile strength did not significantly increase. This is because the PCL chain segments are located at both ends of the GAP chain, leading to increased steric hindrance at the crosslinking points. This hinders contact with other chain segments, and thus, the intermolecular forces in BLOCK elastomers are primarily derived from the GAP chain segments, resulting in little change in cohesive energy compared to GAP elastomers. In contrast, the tensile strength of BLEND elastomers is more than twice that of GAP elastomers due to the incorporation of PCL chain segments into the crosslinked network, leading to greater intermolecular forces attributed to the higher polarity of PCL segments compared to GAP chain segments. However, the elongation at break of BLEND elastomers is reduced by nearly half, likely due to the lower molecular weight of the PCL used in the blends and the increased crosslink density resulting from greater physical entanglements, which reduces the molecular weight between crosslinks.

The GRAFT elastomer exhibits the most optimal overall performance. Its tensile strength is slightly higher than that of the BLEND elastomer, and its elongation at break is only about 10% lower than that of the GAP elastomer. The enhanced performance of the GRAFT elastomer is attributed to the increased number of physical crosslinking points resulting from hydrogen bonding and the formation of crystalline hard segment domains. This improves the crosslinking density of the network structure, thereby enhancing the tensile strength. Low-field NMR results indicate the presence of numerous hard segment regions formed by hydrogen bonding in BLEND elastomers. However, combined with the AFM results, it can be inferred that the hydrogen bonding may be primarily localized within PCL microspheres, contributing less to the overall crosslinked network. While the total number of hydrogen bonds in GRAFT elastomers is lower than that in BLEND elastomers, the microphase separation structure allows the hydrogen bonds to effectively participate in the formation of the crosslinked network. This enables the elastomer to distribute stress throughout the material, improving its mechanical properties. Such improvement is also reflected in the fracture toughness, as illustrated by the critical stress intensity factor (K_IC_) results for the four elastomers in Figure 6c. In the GRAFT elastomer, the presence of microcrystalline regions can impede crack propagation pathways, forcing crack deflection and tortuosity. While the BLOCK elastomer lacks microcrystalline reinforcement, its higher molecular weight between crosslinks (Mc) enhances chain flexibility, facilitating energy dissipation through chain slippage of long PCL segments. The reduced fracture toughness of the BLEND elastomer compared to the CONTROL is likely attributable to phase separation between GAP and PCL. These phase interfaces act as stress concentration sites, where stress accumulates under applied load, accelerating crack initiation and propagation. Scanning electron microscopy (SEM) images of the tensile fracture surfaces of the three elastomers are shown in Figure 6d–f. In contrast to the relatively smooth fracture surfaces observed in the BLEND and BLOCK elastomers, the GRAFT elastomer exhibits numerous ridges and valleys. This rough morphology suggests that the phase-separated microcrystalline structure effectively hinders crack propagation during tensile deformation, thereby positively contributing to the mechanical properties of the elastomer.

### 3.5. Dynamic Mechanical Properties of GAP/PCL Elastomers

**Figure 7:**
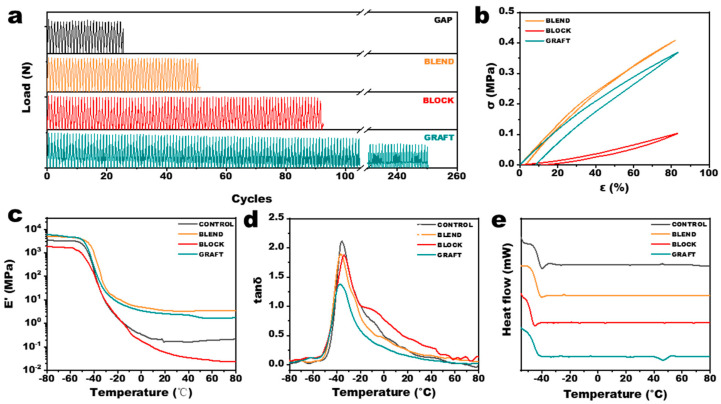
Characterization of GAP/PCL elastomers. (**a**) Fatigue performance, (**b**) cyclic tensile curves, (**c**) storage modules, (**d**) loss factors and (**e**) DSC thermograms.

Cyclic loading tests were performed on the elastomers to evaluate the mechanical durability of the adhesive under simulated ignition/extinction cycles, with the results presented in Figure 7a. The GRAFT elastomer exhibited a significantly higher number of cycles to failure compared to the other three elastomers. This enhanced durability can be attributed not only to the presence of microcrystalline regions that impede crack propagation but, more importantly, to the ability of the elastomer to dissipate energy through the reversible disruption and reformation of hydrogen bonds and microcrystalline regions under cyclic loading. This mechanism effectively suppresses the initiation of microcracks. Similarly, the BLOCK elastomer, which can more readily dissipate energy through chain slippage of long PCL segments, also demonstrated a relatively high number of cycles to failure. The large hysteresis loops observed in the cyclic loading curves of the GRAFT and BLOCK elastomers (Figure 7b) further support this energy dissipation mechanism.

The dynamic mechanical properties are shown in Figure 6d and Figure 7c. The energy storage modulus is higher than that of pure GAP elastomers at all temperatures for both BLEND and GRAFT elastomers. The loss factor (tan δ), defined as the ratio of loss modulus to storage modulus, reflects the intrinsic properties of the propellant through α and β relaxations at the molecular scale. α-relaxation is caused by the motion of the main molecular chains, while β-relaxation is caused by the motion of connecting chains, short chains, branched chains, and other chain segments. The β-relaxation temperature of the BLEND elastomer is similar to that of the GAP elastomer (around −57 °C), while the β-relaxation temperatures of the BLOCK and GRAFT elastomers decrease to −62 °C and −64 °C, respectively. This reduction in β-relaxation temperature can be attributed to the lubricating effect of the flexible PCL linkages on the GAP chain segments. These results suggest that PCL chain segments not chemically bonded to GAP tend to aggregate and do not significantly participate in the crosslinking network during curing. DSC measurements of the elastomers (Figure 7e) are consistent with the XRD results, showing crystalline melting peaks only in the GRAFT elastomer. The greater flexibility of the PCL chain segments results in lower glass transition temperatures (Tg) for all three GAP/PCL elastomers compared to the GAP elastomer, predicting enhanced low-temperature performance. The GRAFT elastomer exhibits the largest decrease in Tg, from −45.6 °C to −48.2 °C, attributed to its branched structure providing more space for chain segment movement.

### 3.6. Thermal Stability of GAP/PCL Elastomers

**Figure 8:**
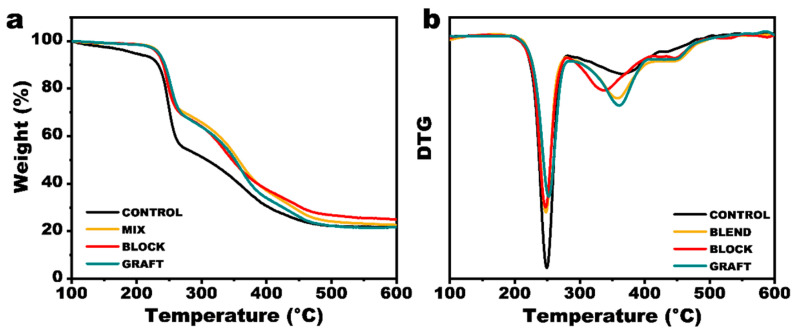
(**a**) TG and (**b**) DTG curves of elastomers.

The decomposition behavior of the binder system plays a crucial role in the combustion characteristics of composite solid propellants, exhibiting a strong correlation with key performance parameters such as heat of explosion, detonation energy, and detonation velocity. To gain a comprehensive understanding of the impact of PCL incorporation on the thermal properties of the binder system, TGA-FTIR was employed to investigate the decomposition behavior of GAP and GAP/PCL elastomers. The results are presented in Figure 8 and Table S2. The thermal decomposition of the GAP elastomer was observed to proceed in three distinct stages. The first stage (208.5 °C to 281.0 °C) corresponds to a mass loss of 42.1%, attributed to the scission of -N_3_ groups on the polymer chains. The second stage (281.0 °C to 424.0 °C) exhibits a mass loss of 26.5%, primarily associated with the thermal decomposition of the curing agent and reaction products such as urethane, ureaurethane, and biuret groups. Subsequently, the decomposition of the GAP backbone occurs. At 600 °C, the residual char yield was 25.2%. Compared to the neat GAP elastomer, the GAP/PCL elastomer exhibited an increase in the initial decomposition temperature, indicating that PCL may provide a protective barrier, hindering heat transfer to the azido groups and enhancing the thermal stability of the GAP film. Furthermore, the introduction of PCL introduces ester carbonyl groups, which are more susceptible to decomposition than urea carbonyl groups. Concurrently, FTIR analysis (Figure S7) reveals that PCL undergoes initial decomposition, generating a substantial amount of CO_2_, which may exert an inhibitory effect on free radical chain reactions. These two factors contribute to a significant reduction in the decomposition temperature of the second stage in the GAP/PCL elastomer, potentially improving the controllable combustion of the propellant.

## 4. Conclusions

In this study, PCL-b-GAP-b-PCL with a block structure and GAP-g-PCL with a grafted structure were successfully synthesized. Characterization tests confirmed the synthesis of the target GAP/PCL structures. The grafted structure led to greater spacing between molecular chains, resulting in a lower viscosity for GAP-g-PCL compared to PCL-b-GAP-b-PCL. Additionally, DSC analysis revealed no crystallization in PCL-b-GAP-b-PCL, attributed to the short PCL segment length. The objective of this study was to prepare elastomers from these synthetic GAP/PCL prepolymers via polyurethane curing and to compare them to GAP elastomers produced by physical blending. The results show that in BLEND elastomers, increased tensile strength is achieved through a higher number of physical crosslinking points. However, significant PCL chain precipitation prevents their effective integration into the crosslinked network. BLOCK elastomers exhibit enhanced elongation at break but possess fewer chemical and physical crosslinking points, resulting in relatively low tensile strength. In contrast, GRAFT elastomers, without PCL precipitation, form a more effective tensile network due to the longer PCL chain segments and increased chain segment mobility. The formation of microcrystalline regions enhances physical crosslinking and inhibits microcrack propagation, significantly improving the elastomer’s mechanical properties. DSC and TGA results demonstrate that GAP/PCL elastomers have lower glass transition temperatures than pure GAP elastomers, indicating improved low-temperature performance, and show increased thermal stability. Among all samples, the GRAFT elastomer exhibited the most substantial combined performance enhancement, with a more-than-twofold increase in tensile strength despite only a ~15% reduction in elongation at break. The GRAFT elastomer also showed a notable decrease in Tg from −45.6 °C to −48.2 °C and a slight decrease in initial decomposition temperature of 3.3 °C. This research provides a novel approach and a solid experimental basis for incorporating flexible-chain modification into GAP-based solid propellants.

## Data Availability

Data are contained within the article and Supplementary Materials.

## Supplementary Materials

The following supporting information can be downloaded at: https://www.mdpi.com/article/10.3390/polym17050661/s1, Figure S1: The synthetic route of PCL-b-GAP-b-PCL (a) and GAP-g-PCL (b); Figure S2: FTIR spectra of PCL and ALk-PCL; Figure S3: NMR spectra of GAP and Alk PCL at different reaction stages; Table S1: Elastomerr information; Figure S4: FTIR spectra of different elastomers; Figure S5: XRD spectra of GAP elastomers blended with PCL of different molecular weights and mass fractions; Table S2: Thermal stability parameters of elastomers; Figure S6: Crosslinking density of elastomers measured by low field nuclear magnetic resonance; Figure S7: Three-dimensional FTIR spectra of the evolved gases during the thermal decomposition of elastomers.
