# Supplementary material for: Effect of Poly (Caprolactone) Introduction Site on the Network Structure and Properties of Glycidyl Azide Polymer Adhesive
Source: Polymers (Basel). 2025 Feb 28;17(5):661. doi: 10.3390/polym17050661 (PMC11902306; doi:10.3390/polym17050661)
Supplement: Supplementary file 1 [file polymers-17-00661-s001.zip › polymers-3460503-supplementary.pdf]

polymers

Support Information

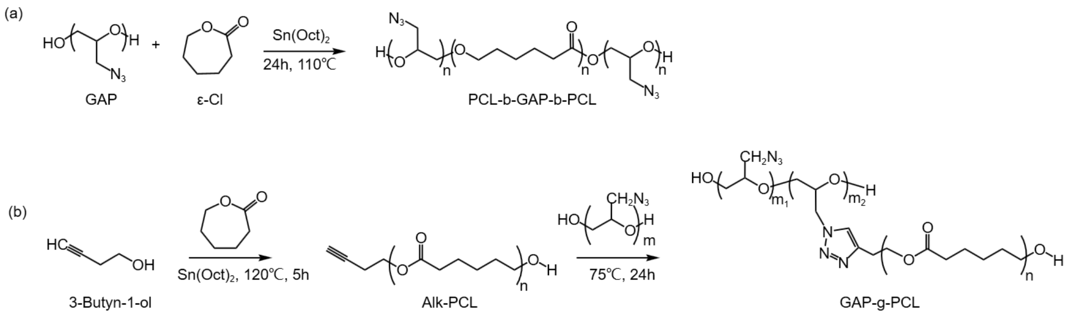

Figure S1. The synthetic route of PCL-b-GAP-b-PCL (**a**) and GAP-g-PCL (**b**).

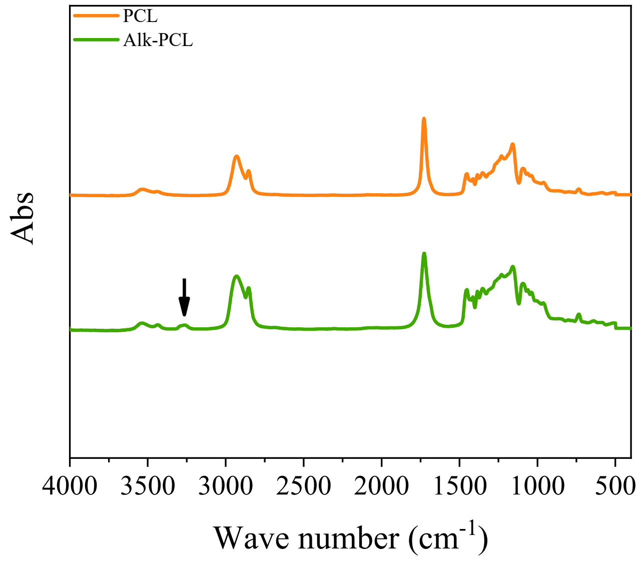

Figure S2. FTIR spectra of PCL and ALk-PCL.

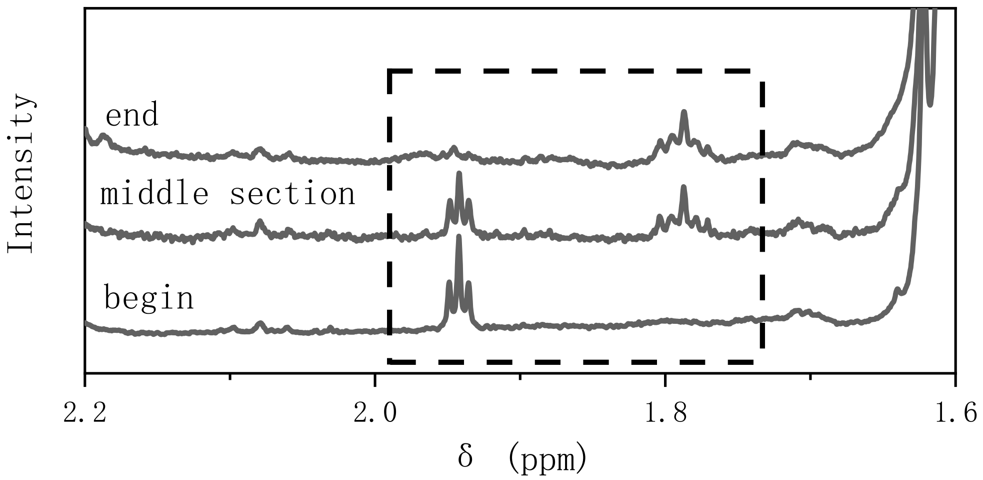

Figure S3. NMR spectra of GAP and Alk PCL at different reaction stages.

Table S1. Elastomerr information.

	Prepolymer	Curing Agent	R Value
CONTROL	GAP	N-100	1.2
BLEND	GAP&PCL		
BLOCK	PCL-b-GAP-b-PCL		
GRAFT	GAP-g-PCL		

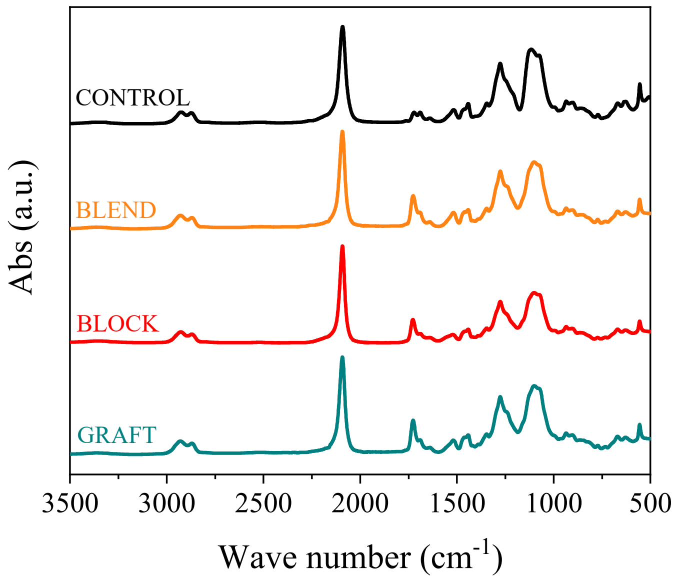

Figure S4. FTIR spectra of different elastomers.

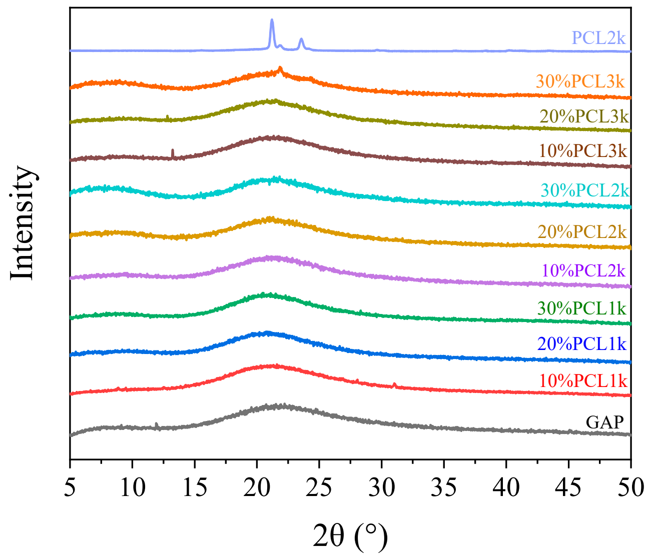

Figure S5. XRD spectra of GAP elastomers blended with PCL of different molecular weights and mass fractions.

Table S2. Thermal stability parameters of elastomers.

Sample	$T_{5\%}$/°C	T_{MAX}/°C	
		T_{MAX1}	T_{MAX2}
CONTROL	230.5	248.9	358.0
BLEND	234.4	247.6	337.3
BLOCK	231.9	247.3	413
GRAFT	233.8	257.1	412

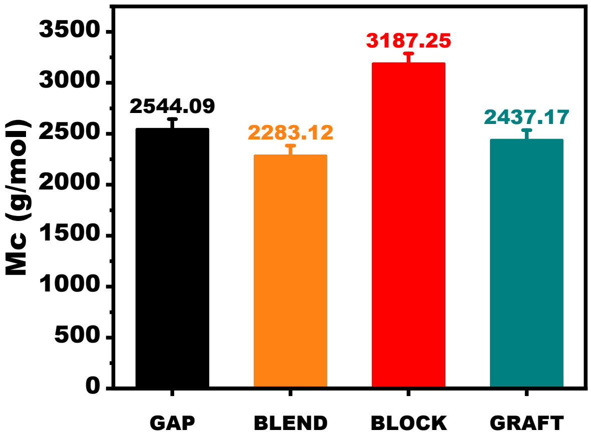

Figure S6. Crosslinking density of elastomers measured by low field nuclear magnetic resonance.

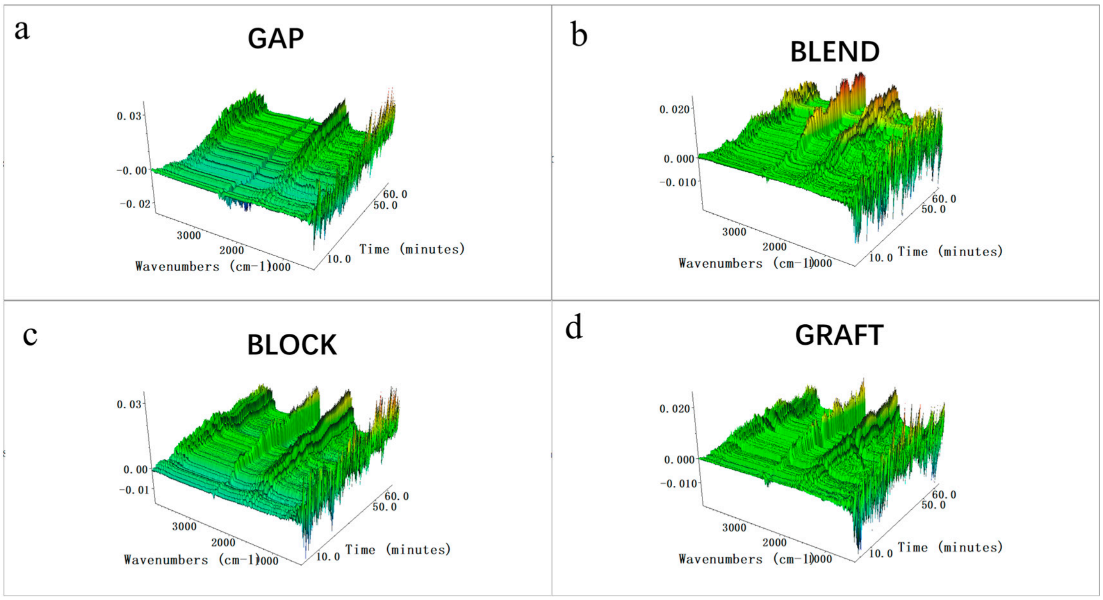

Figure S7. Three-dimensional FTIR spectra of the evolved gases during the thermal decomposition of elastomers.
